# Simple ClinVar: an interactive web server to explore and retrieve gene and disease variants aggregated in ClinVar database

**DOI:** 10.1093/nar/gkz411

**Published:** 2019-05-22

**Authors:** Eduardo Pérez-Palma, Marie Gramm, Peter Nürnberg, Patrick May, Dennis Lal

**Affiliations:** 1 Cologne Center for Genomics, University of Cologne, 50939 Cologne, NRW, Germany; 2 Luxembourg Centre for Systems Biomedicine, University Luxembourg, L-4362 Esch-sur-Alzette, Luxembourg; 3 Epilepsy Center, Neurological Institute, Cleveland Clinic, 44195 Cleveland, OH, USA; 4 Genomic Medicine Institute, Lerner Research Institute, Cleveland Clinic, 44195 Cleveland, OH, USA; 5 Stanley Center for Psychiatric Research, Broad Institute of MIT and Harvard, 02142 Cambridge, MA, USA

## Abstract

Clinical genetic testing has exponentially expanded in recent years, leading to an overwhelming amount of patient variants with high variability in pathogenicity and heterogeneous phenotypes. A large part of the variant level data is aggregated in public databases such as ClinVar. However, the ability to explore this rich resource and answer general questions such as ‘How many genes inside ClinVar are associated with a specific disease? or ‘In which part of the protein are patient variants located?’ is limited and requires advanced bioinformatics processing. Here, we present Simple ClinVar (http://simple-clinvar.broadinstitute.org/) a web server application that is able to provide variant, gene and disease level summary statistics based on the entire ClinVar database in a dynamic and user-friendly web-interface. Overall, our web application is able to interactively answer basic questions regarding genetic variation and its known relationships to disease. By typing a disease term of interest, the user can identify in seconds the genes and phenotypes most frequently reported to ClinVar. Subsets of variants can then be further explored, filtered or mapped and visualized in the corresponding protein sequences. Our website will follow ClinVar monthly releases and provide easy access to ClinVar resources to a broader audience including basic and clinical scientists.

## INTRODUCTION

In recent years, clinical genetic testing has expanded exponentially alongside transparent and persistent data sharing. ClinVar is a public database of variant interpretations (1) that has steadily grown to become the largest publicly available genetic variant database and provides an ever-growing resource to study genotype–phenotype correlations. The database is populated by a broad range of submitters including but not limited to researchers, clinicians, laboratories and genetic counselors (1). However, most data are derived from clinical genetic testing laboratories. Since its inception in 2013, ClinVar has grown rapidly (https://www.ncbi.nlm.nih.gov/clinvar/). As of 22 April 2019, ClinVar contains 503 065 unique genetic variants from 1229 submitters from all around the world. As a consequence, ClinVar has become a valuable resource to support clinical variant interpretation (2).

ClinVar is maintained by the National Center for Biotechnology Information (NCBI) and is tightly connected to multiple key NCBI resources such as dbSNP (3), PubMed Central (4) or the Reference Sequence Database (5). Alongside the genetic variants and the associated phenotypes, ClinVar provides for each variant entry more than 30 fields of data that come in multiple levels and are connected to external resources. Furthermore, it provides clinical consequence interpretations (i.e. Pathogenicity levels) as well as reliability categories such as the ‘Review status’ (e.g. single or multiple submitters).

While ClinVar stands out as one of the largest clinical genetic resource available due to its volume and complexity, it also introduces limitations to the user experience. For example, a single search yields a multiple page table with a subset of variant features led by the variant name in Human Genome Variation Society (HGVS) sequence variant nomenclature format (6). The HGVS format is difficult to read by non-experts. To access features such as variant type (e.g. duplications, single nucleotide variant) and molecular consequence (e.g. missense and nonsense variants) the user needs to be redirected to another variant level page. A basic user will need to interrogate variants one by one with multiple searches. Without prior bioinformatics knowledge, the evaluation of the entire database, filtering, or parsing of multiple high confidence variants becomes extremely difficult. To address this issue, several platforms have been developed such as ClinVar Miner (7), ClinVar data parsing (8) and Clinotator (9) that offer parsing solutions limited to the extraction of ClinVar variants and the development of additional interpretation metrics (9) that can be still overwhelming for non-expert users. In this regard, ClinVar filtering is often not enough to facilitate user analysis. Redundant or contradictory collapsed fields such as clinical significance (e.g. ‘Likely benign’ alongside ‘Risk’ evidence) and phenotype annotation (e.g. ‘not-provided’ alongside ‘not specified’) can often lead to confusion. Additionally, graphical and dynamic visualization of ClinVar's main features is not provided. Genetic variants are given without the context of their location over the entire protein sequence. For example, with the available tools, it is not possible to determine if a stop codon variant occurs near the end of the protein sequence or if a missense variant falls within the boundaries of a functional domain. To address these needs, we developed the Simple ClinVar web server (http://simple-clinvar.broadinstitute.org) that provides extraction and filtering of ClinVar entries together with a dynamic graphical visualization of ClinVar data in an intuitive way. Simple ClinVar complements the existing ClinVar analysis by enabling exploration of the data at different levels of granularity and access to recalculated summary statistics for current data. Here, we describe the functionality of the components of Simple ClinVar and demonstrate the use of the tool to investigate the ClinVar database.

## MATERIALS AND METHODS

### ClinVar pre-filtering

**Figure 1.**
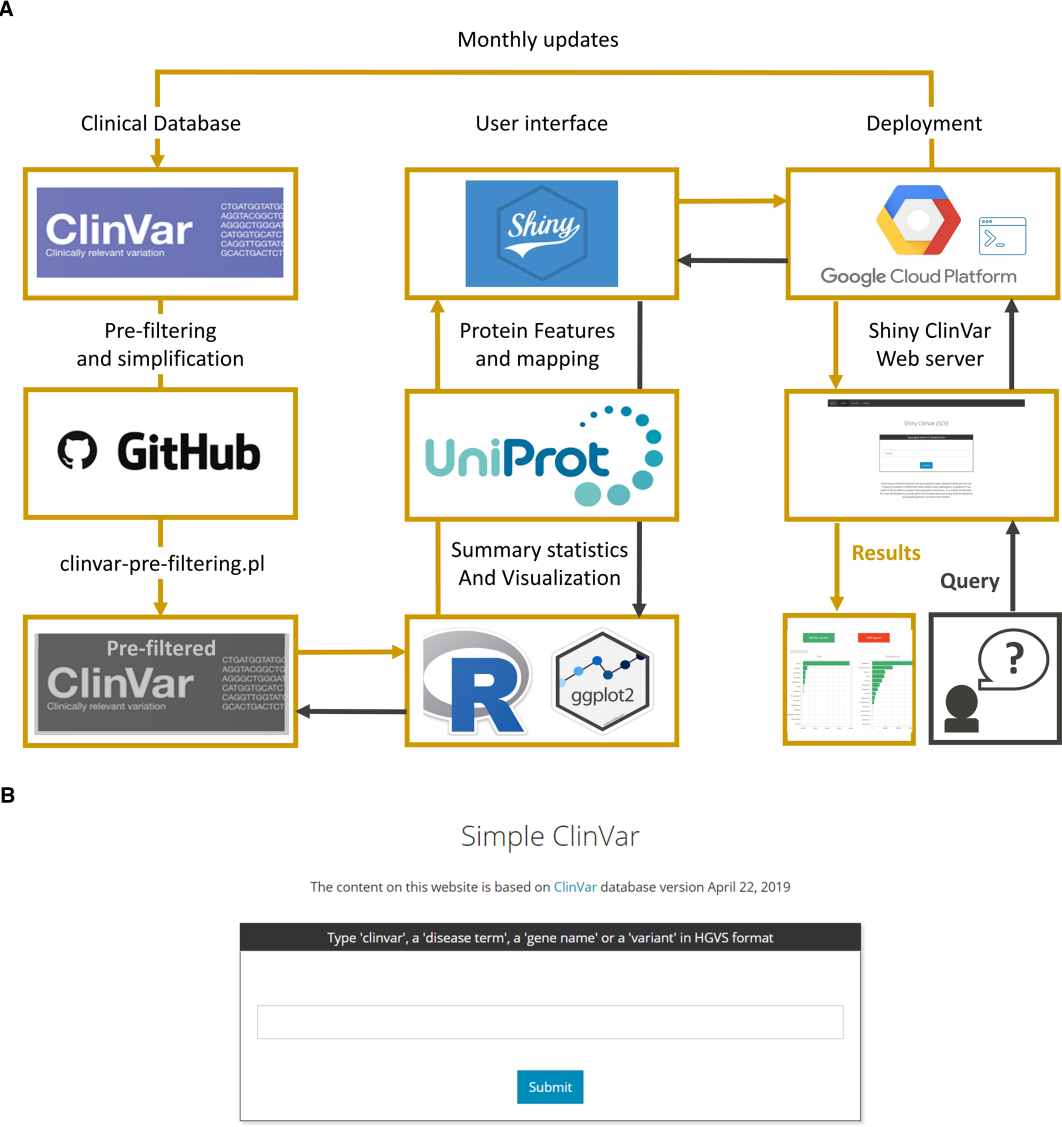
Simple ClinVar internal workflow and main module. (A) Information flow overview of the Simple ClinVar web application. The ClinVar database is pre-filtered with the clinvar.pre-filtering.pl script available at our GitHub page (http://github.com/dlal-group/Simple-ClinVar) to generate the ClinVar prefiltered file that is interrogated by the user. Upon query submission, the user interface generated with R software connects with pre-filtered file and UniProt features to deliver a result. (B) Simple ClinVar front page where the user can perform different types of queries.

The ClinVar database (ClinVar) is downloaded monthly in tab-delimited format (10) directly from the ftp site (ftp://ftp.ncbi.nlm.nih.gov/pub/clinvar/tab_delimited/variant_summary.txt.gz). The tabular data contained in the variant_summary.txt.gz file is processed internally to produce a pre-filtered ClinVar file for the user to explore in the Simple ClinVar web server (Figure 1A). First, we keep only entries from the human reference genome version GRCh37.p13/hg19 referring to canonical transcripts. Reference canonical transcripts were extracted from the Ensembl VEP tool version 96 (11). Second, ‘molecular consequence’ is inferred through the analysis of the HGVS sequence variant nomenclature field (6). Third, we reduce the complexity of the' clinical significance' field by regrouping and merging them into five unique categories: ‘Pathogenic’, ‘Likely pathogenic’, ‘Risk factor and Association’, ‘Protective/Likely benign’ and ‘Benign’. Additional categories, namely: conflicting interpretations of pathogenicity, variants of unknown significance (VUS) and variants with contradictory evidence share an inconclusive interpretation status. Accordingly, we combine these into a unique ‘Uncertain/Conflicting’ category. Similarly, variants annotated with multiple evidence categories of the same evidence direction such as ‘Pathogenic’ alongside ‘Likely pathogenic’ are combined and the respective lower evidence category is assigned. Fourth, ClinVar entries with phenotypes annotated as ‘not provided’ and ‘not specified’ are combined into one single category called ‘Not provided/Not specified’. ClinVar entries with missing annotations such as the absence of an HGVS variant name or incomplete genomic coordinates are filtered out. By the time of submission 493 240 out of 503 065 (98.04%) ClinVar entries (April 22 release) are included in Simple ClinVar. The output constitutes the pre-filtered ClinVar file. The complete processing pipeline is implemented in a single Perl script (clinvar.pre-filtering.pl) available at our GitHub repository (http://github.com/dlal-group/Simple-ClinVar).

### Simple ClinVar web server development

Interactive summary statistics, variant mapping and visualization provided by the Simple ClinVar web server are based on the entries available in the pre-filtered ClinVar file and were developed with the Shiny framework of R studio software (https://shiny.rstudio.com/) which transforms regular R code into an interactive environment that is able to follow and ‘react’ to remote-users instructions. In the background, Shiny translates user-queries into HTML code which can, in turn, be displayed by any web browser. Pre-filtered ClinVar file alongside the R/Shiny code was uploaded as a self-standing Ubuntu 16.04 LTS image with Google Cloud services. The image was deployed into a Google Virtual Machine (VM) using the googleComputeEngineR package (https://github.com/cloudyr/googleComputeEngineR). The Simple ClinVar web server (http://simple-clinvar.broadinstitute.org/) is compatible with all commonly used internet browsers including mobile devices versions. Simple ClinVar will extract all the entries (rows) from the file that contains a user query (e.g. disease term or gene HGNC symbol) in the corresponding column (genes, variants or phenotype). From that subset of rows, it will perform the calculations and visualization. The visualization of protein sequences together with their domain boundaries was developed with the R drawProteins package (https://github.com/brennanpincardiff/drawProteins) which queries the information directly from UniProt database (12) (UniProt release 2019_03). All graphs shown in the present work and generated by the web server are based on the ggplot2 R library and plotly R library (https://plot.ly/r/).

## RESULTS

### Simple ClinVar web server

Simple ClinVar works over the pre-filtered version of the ClinVar database. The processed file is designed to reduce the complexity of ClinVar entries as well as to provide fast access to high-quality entries coupled with additional features such as protein domain annotations and variant mapping (Figure 1A). The ClinVar database is constantly growing. As such, the database is updated on a regular basis and released to the public. Processing of the latest ClinVar version to update Simple ClinVar is performed monthly. From the front page of Simple ClinVar, the user can submit three types of queries. First, the database-wise query shows the total counts of variants, genes, and phenotypes available in the current ClinVar database. Second, the gene-wise query lists variant counts and associated diseases for every entered gene. Here, the user can visualize the extracted variants on the encoded protein sequence to evaluate variant distribution by variant type across the protein sequence. Third, the disease term query provides the user with all genes, variants, and phenotypes associated with a given disease term.

### Database-wise query (Figure 2A)

Fast and user-friendly evaluation of the full information available in the entire pre-filtered ClinVar file is possible with the database-wise query. Triggered by submitting without a query or with the keyword ‘clinvar’, it will yield summary statistics of the entire ClinVar database. By the time of submission (ClinVar 22 April 2019 release) Simple ClinVar contains 493 240 genetic variants, identified in 18 502 genes found in patients with 11 098 phenotypes. The database query mode coupled with dynamic filtering allows the user to explore which are the most common disorders and types of variants most commonly found in the whole database. Similarly, it is possible to evaluate immediately which are the genes with the most pathogenic variations or VUS.

### Gene-wise query (Figure 2B)

The main advantage of this query type is that variants can be mapped over protein sequences. Submitting a HGNC gene symbol on the main page will forward the user to the corresponding summary statistic page for all the genetic variants annotated in the gene. For example, querying for ‘*CDKL5*’ will show 675 genetic variants currently associated with 27 genetic disorders. Here, the user will see these variants mapped over the corresponding protein sequence alongside domain information from UniProt. For densely annotated regions or large proteins, the user can zoom in over the protein sequence to improve visualization. Furthermore, the user can explore unique gene-specific variant statistics such as determining how many clinical phenotypes are associated with a given gene or where the pathogenic versus benign variants are located in the protein.

### Disease-term-wise query (Figure 2C)

Disease-oriented queries are particularly limited in ClinVar. To answer which are the top 10 genes associated with ‘heart disease’ is not straightforward even with available ClinVar related tools. One of the biggest challenges is that the clinical syndromes are annotated in a non-uniform way and it is not clear to the user which terms are most frequently used. Querying a broad disease term of interest in Simple ClinVar allows the identification and extraction of the most used phenotype terms and disorder subtypes for disease annotated in ClinVar. As an example, the disease term query ‘heart’ will yield 814 genetic variants in 61 genes associated with 233 phenotypes, with missense SNVs (*n* = 321) as the most common variant type. Here, for each selected disease term the user can answer general questions such as: how many genes are associated with heart disease? How many annotated terms and disorder subtypes can be found related to heart disease?.

At last, if the user is interested in querying a single ClinVar variant we have enabled a ‘variant-wise’ search that allows the user to query for single variants in HGVS format (e.g. NM_021007.2(SCN2A):c.5620G>A (p.Ala1874Thr)). The ‘variant-wise’ result section will display summary statistics, variant mapping over the affected gene and phenotypes associated with it in the same format displayed for the other types of inputs.

### Organization and visualization of the results page

**Figure 2.**
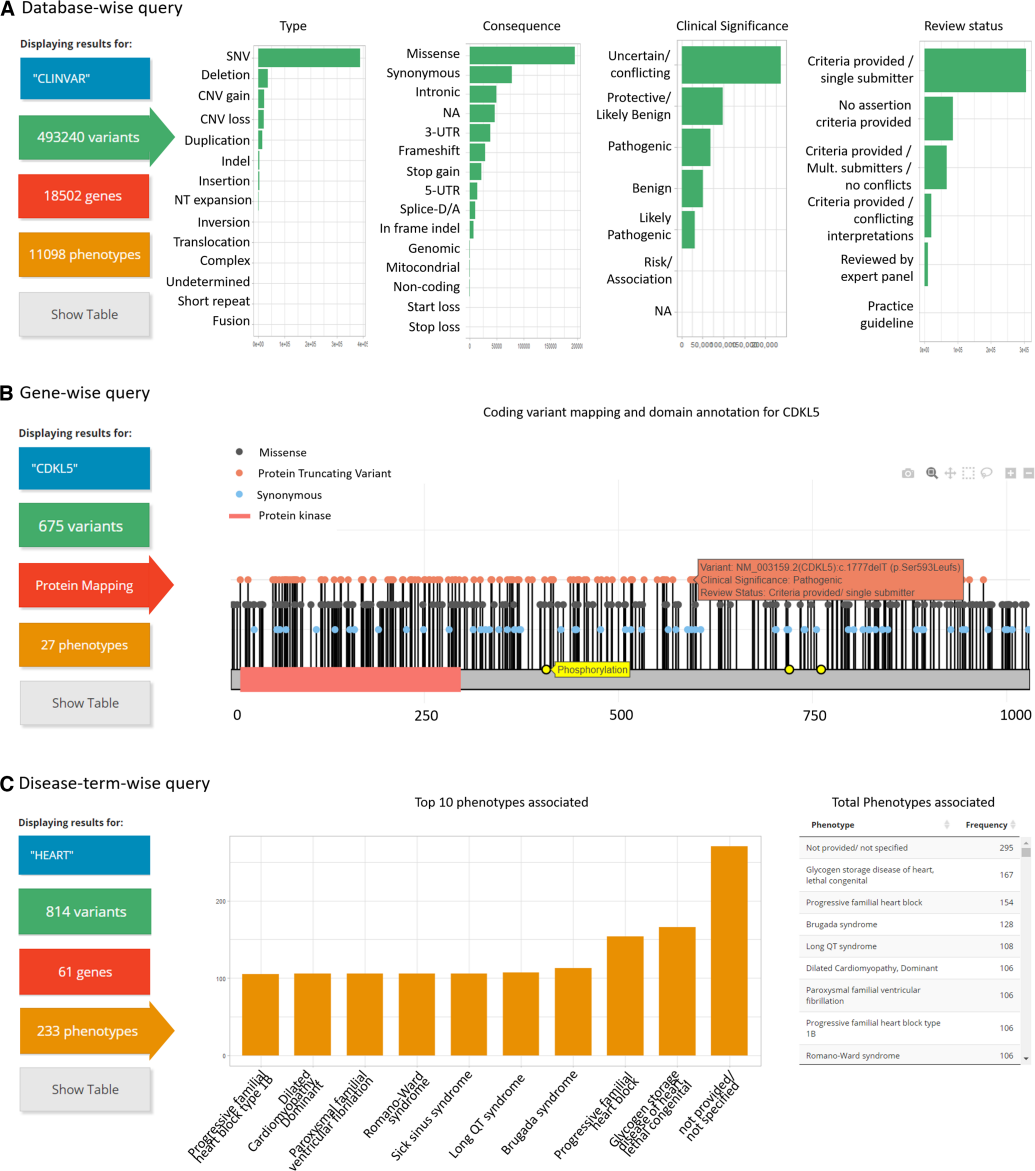
Simple ClinVar results section according to the three types of input supported. Examples of Simple ClinVar outputs according to (**A**) Database-wise query, (**B**) Gene-wise query and (**C**) Disease-term-wise query. The user can dynamically change between variant view (green, A), gene view (red, B), phenotype view (orange, C) and the table view (gray). For gene view, variants positions are shown according to the corresponding protein sequence. Here, if the user place the cursor over a variant a tooltip will appear with summary information (orange rectangle). Genetic variants are colored in green for synonymous, gray for missense, and red for protein truncating variants (Frameshift, small indels and stops gained).

Independently of the input mode, the output displayed at the results tab can always be explored between four sections marked by the top square buttons in the colors green, red, orange and gray (Figure 2, left panel). The user can switch between the color areas. The green button will show all genetic variants available for the query and see the counts of variant type, molecular consequence, clinical significance and review status. The red and orange buttons will show the top 10 genes and phenotypes associated with the corresponding query and the complete list in table mode, respectively. In the case of a gene-wise or variant-wise query submission, the red button will show the variant mapping over the canonical protein sequence of the gene queried. At this level, hovering over each variant mapped will display a tooltip with the variant main features, namely the variant name in HGVS format, clinical significance, and review status. Click on any variant will activate hyperlink to the corresponding entry in ClinVar database. At last, the gray button will activate the table mode where the subset of the pre-filtered ClinVar entries currently in display is shown. All tables shown are available for download. Table view first column contains ClinVar variant identifier hyperlinked to ClinVar Database for further exploration.

### Practical filtering examples

**Figure 3.**
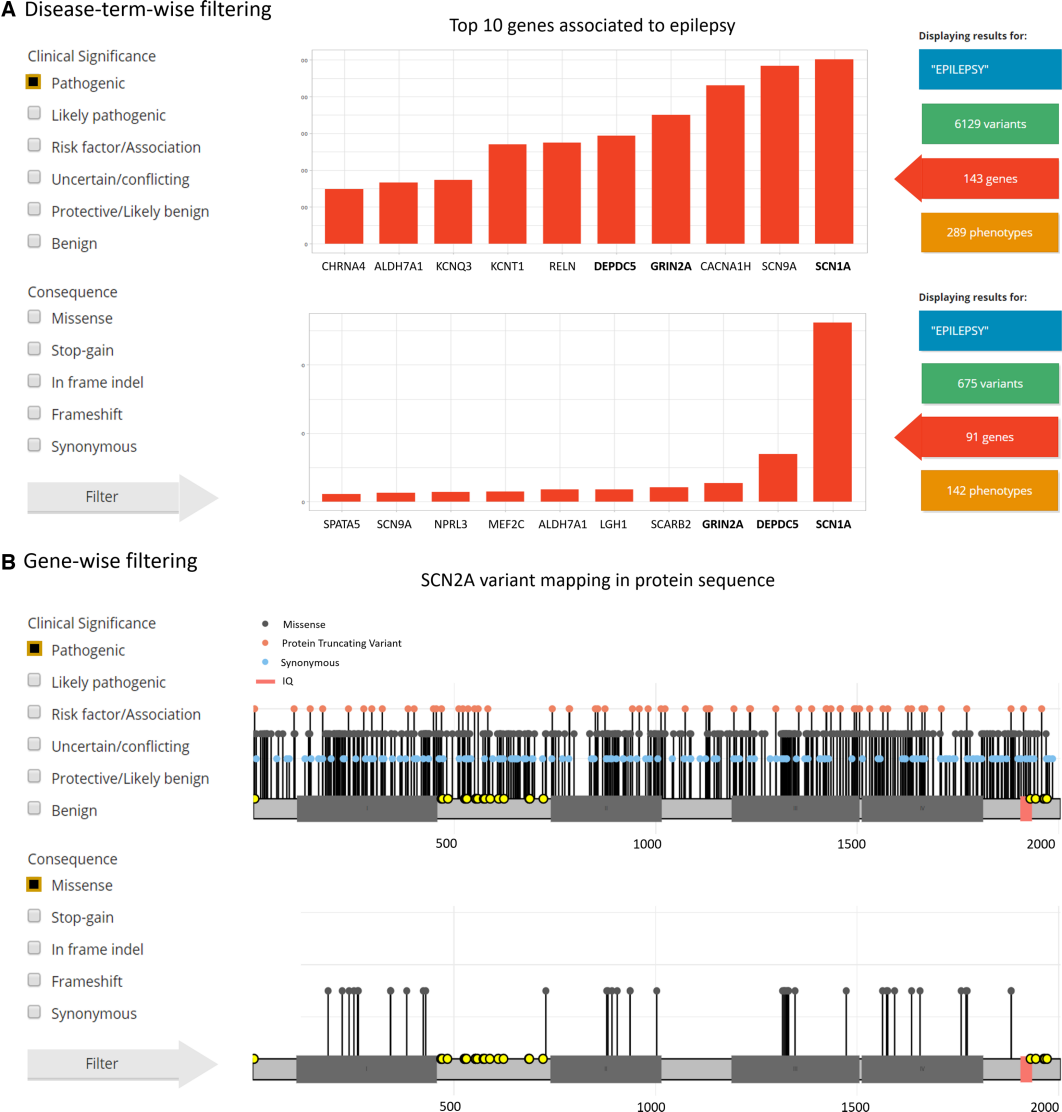
Dynamic filtering examples. (**A**) Gene view of the Disease-term-wise query for ‘epilepsy’ shows all genes associated before (upper panel) and after (bottom panel) filtering for pathogenic variants (left panel). (**B**) Gene view of the gene-wise query ‘SCN2A’ shows the genetic variants mapped on the protein sequence before (upper panel) and after (bottom panel) filtering for pathogenic and missense variants (on the left).

At all query levels, the output can be dynamically filtered by variant type, molecular consequence, clinical significance and review status in any combination. We show two examples of how this feature can be used as a fast and powerful tool for clinical researchers. First, we use ‘epilepsy’ as a disease term query and display the red button gene view area (Figure 3). Unfiltered results show the top 10 ‘epilepsy’ genes associated in descending order according to the number of qualifying variants: *SCN1A, SCN9A, CACNA1H, GRIN2A, DEPDC5, RELN, KCNT1, KCNQ3, ALDH7A1* and *CHRNA4* (Figure 3A, top panel). Next, after filtering for pathogenic variants, the top 10 gene list is updated to *SCN1A, DEPDC5, GRIN2A, SCARB2, ALDH7A1, LGI1, MEF2C, NPRL3, SCN9A* and *SPATA5*. The user can conclude that the order of frequently mutated genes and genes with the most pathogenic classified genes is not the same. In the example, only *SCN1A, DEPDC5* and *GRIN2A* are in the top 10 ‘epilepsy’ gene list both as genes with the most variants and most pathogenic variants (Figure 3A, lower panel). Second, we evaluate the gene-wise query for ‘SCN2A’ on the red button gene view (Figure 3B, upper panel). By the time of submission, there were 678 variants mapped on the protein sequence of *SCN2A*. If we filter for ‘Missense’ and ‘Pathogenic’ only 40 variants remain and are concentrated inside the transmembrane domains (Figure 3B, lower panel). The user can conclude that these regions containing the majority of pathogenic variants are of key importance for the protein function.

## DISCUSSION AND FUTURE DIRECTIONS

ClinVar stands out today as one of the largest catalogs of genetic variants clinically associated with disease. Nevertheless, given its volume and complexity, easy access to the information available is not entirely achievable through the database platforms or associated tools. Here, we have developed Simple ClinVar, a novel web application that provides fast variant, gene, and phenotypes level summary statistics from the ClinVar database, interactively displayed in a user-friendly interface.

Using a single query form, users can answer basic questions regarding genetic variation and their known relationships to disease from three different views: database-wise, gene-wise and disease-term-wise. Furthermore, the application is designed to work on multiple browser platforms including mobile devices, which becomes particularly useful for quick queries needed while attending scientific meetings or reading scientific publications. Filtering by variant type, molecular consequence, clinical significance and review status provides the user with novel research opportunities such as identifying a cluster of pathogenic variants over protein domains or comparing their distribution with lower confidence variants. Clinical research questions can also be addressed, such as identifying whether the most frequent disease genes reported in ClinVar are also the most frequent positives in clinical genetic testing or whether a particular gene panel includes the most commonly or recently associated genes. Simple ClinVar provides a unique and novel framework that is capable of answering these types of questions in less than a minute. It is widely acknowledged that the ClinVar database is growing exponentially and that constant revisiting of low confidence entries (1) is making the available information increasingly more reliable. Monthly updates will enable a stable follow-up of these changes.

Simple ClinVar's framework currently has some limitations. For example, users interested in variant interpretation may want to investigate if the patient variants currently in display on Simple ClinVar have been observed in the general population (13). To address this issue, we plan for future Simple ClinVar releases to include a direct comparison with variants from the genome aggregation Database (gnomAD) public release 2.0.2 (14). Additionally, since potential users may have their own set of variants not yet present in ClinVar we aim to develop an upload option to map and compare user-defined variants with variants present in ClinVar. Currently, the Simple ClinVar framework can potentially be linked to other variant repositories such as HGMD (15) or LOVD (16). Advanced users can implement the pre-filtering pipeline (available at http://github.com/dlal-group/Simple-ClinVar) and then run Simple ClinVar locally and independently summarize and visualize their own genetic clinical data.

Overall, the Simple ClinVar application will grow in size and accuracy alongside future ClinVar releases. Simple ClinVar will expedite genetic inquiries for researchers and clinicians as well as provide an intuitive and helpful tool to bring the growing breadth of genetic knowledge to a broader audience of clinicians and researchers with little or no prior bioinformatics expertise.
